# Effects of fMRI neurofeedback of right inferior frontal cortex on inhibitory brain activation in children with ADHD

**DOI:** 10.1098/rstb.2023.0097

**Published:** 2024-10-21

**Authors:** Steve Lukito, Sheut-Ling Lam, Marion Criaud, Samuel Westwood, Olivia S. Kowalczyk, Sarah Curran, Nadia Barrett, Christopher Abbott, Holan Liang, Emily Simonoff, Gareth J. Barker, Vincent Giampietro, Katya Rubia

**Affiliations:** ^1^ Department of Child and Adolescent Psychiatry, Institute of Psychiatry Psychology & Neuroscience, King’s College London, London, UK; ^2^ School of Biomedical Engineering & Imaging Sciences, King’s College London, London, UK; ^3^ Institute for Globally Distributed Open Research and Education (IGDORE), Gothenburg, Sweden; ^4^ Department of Psychology, Institute of Psychiatry Psychology and Neuroscience, King’s College London, London, UK; ^5^ Department of Neuroimaging, Institute of Psychiatry Psychology and Neuroscience, King’s College London, London, UK; ^6^ South London and Maudsley NHS Foundation Trust, London, UK; ^7^ Tavistock and Portman NHS Foundation Trust, London, UK; ^8^ Great Ormond Street Hospital for Children NHS Foundation Trust, London, UK; ^9^ Department of Child and Adolescent Psychiatry, Technical University, Dresden, Germany

**Keywords:** ADHD, stop-signal task, inhibition, fMRI neurofeedback, children

## Abstract

We aimed to replicate previous effects of functional magnetic resonance imaging neurofeedback (fMRI-NF) in right inferior frontal cortex (rIFC) on IFC activation during a Stop Task in a larger group of boys with attention-deficit/hyperactivity disorder (ADHD). The present double-blind, randomized controlled trial tested the effects of 15 runs of active versus sham fMRI-NF of rIFC on performance and activation associated with successful and failed inhibition versus Go trials during a tracking Stop task in 88 boys with ADHD (44 active; 44 sham), controlling for age and medication status. No significant group-by-time interaction effects were observed for performance or brain activation during the successful stop trials, and post hoc analysis showed very low numbers of active fMRI-NF learners. Nevertheless, during error monitoring, there was a significant group-by-time interaction effect on post-error reaction time slowing and in left IFC activation, which were both increased after active compared to sham fMRI-NF. The findings are in line with our previous observation of left IFC upregulation after fMRI-NF of rIFC relative to active fMRI-NF of parahippocampal gyrus. This highlights the potentially wider regional effects that fMRI-NF of a particular self-control target region has on other self-regulatory regions in ADHD.

This article is part of the theme issue ‘Neurofeedback: new territories and neurocognitive mechanisms of endogenous neuromodulation’.

## 1. Introduction

Attention-deficit/hyperactivity disorder (ADHD) is characterized by persistent, developmentally inappropriate and impairing symptoms of inattention and/or hyperactivity/impulsiveness [1] and is one of the most prevalent (5–7%) neurodevelopmental conditions [2]. ADHD has been associated with deficits in executive functions [3,4], with one of the most consistent deficits being problems with motor inhibitory control as measured in Go/No-go and Stop tasks [5,6]. Furthermore, meta-analyses of functional magnetic resonance imaging (fMRI) studies show consistent underactivation in fronto-striato-thalamic and fronto-parieto-cerebellar regions and networks in ADHD [7–11] and underactivation in right inferior frontal cortex (rIFC) during tasks of inhibitory control is one of the most consistent findings [7–9]. In addition, psychostimulant medication, which is the first-line pharmacological treatment for ADHD [12], has been shown to consistently increase rIFC activation based on a meta-analysis of stimulant medication effects in fMRI studies in children and adults with ADHD [13]. However, stimulants are not indicated for all children with ADHD, are associated with side effects [12], and treatment adherence may be poor in adolescents [14]; further, evidence for their long-term efficacy is limited [12,15], which may be related to brain adaptation to stimulant medication [16].

fMRI-based neurofeedback (fMRI-NF) presents a potential novel alternative to pharmacological treatment for ADHD. Based on operant conditioning, fMRI-NF enables individuals to self-regulate activation in specific brain regions/networks by providing feedback on brain activity in real-time [17–19]. Electroencephalography neurofeedback (EEG-NF) has shown small and non-significant effects in this population in recent meta-analyses [19]. However, compared to EEG-NF, fMRI-NF requires fewer sessions [18,19] and has the advantage that it can target the deeper cortical and subcortical regions that have been shown to be reduced in activation based on the last decade of fMRI studies in ADHD, such as the IFC and the basal ganglia [7–9].

We have previously conducted two pioneering fMRI-NF studies in children and adolescents with ADHD. In the first proof-of-concept single-blind randomized controlled trial (RCT) of fMRI-NF in ADHD [20], ADHD boys were able to progressively increase activation of either rIFC (active group; *n* = 18) or left parahippocampal gyrus (PHG; active control group; *n* = 13), after four 1 h sessions of 11 runs of fMRI-NF with a significant transfer effect in the rIFC group. This was significantly associated with improved ADHD symptoms in both groups relative to baseline, with no side or adverse effects. Although groups did not differ in clinical or cognitive measures, at follow-up, the clinical improvement was more pronounced in the rIFC group (Cohen’s *d~*1), while it was no longer significant in the control group, suggesting potential delayed consolidation or plasticity effects [20]. The most pronounced group differences, however, were observed in fMRI activation during a tracking Stop-signal task. The rIFC fMRI-NF group, compared to the left parahippocampal fMRI-NF group, showed significantly increased rIFC and precuneus activation during successful inhibition [20] and increased left IFC activation during error monitoring after compared to before treatment, which was furthermore correlated with treatment-related ADHD symptom changes [21]. This was accompanied by increased functional connectivity in IFC-cingulo-striatal networks and decreased connectivity of rIFC with areas of the default-mode network [22]. Interestingly, similar upregulation and even normalization effects have been observed in the same regions with stimulant medication relative to placebo, using the same Stop task [13,23,24]. This suggests that fMRI-NF of the rIFC has similar activation effects on the disorder as stimulant medication.

To overcome the limitations of the initial proof-of-concept RCT, such as small sample size, single-blindness and the lack of a placebo condition, we conducted a larger double-blind, sham-controlled RCT in ADHD boys. We tested the efficacy of 15 runs of 7.5 min active fMRI-NF versus sham fMRI-NF of rIFC over four 1 h sessions in a range of clinical, cognitive and Stop task fMRI measures in 88 ADHD boys. We found mostly no significant group differences in clinical or cognitive measures [25]. While the active group, relative to the sham group, showed enhanced activation in rIFC and other frontal and temporo-occipito-cerebellar self-regulation areas, there was no progressive rIFC upregulation across runs, no correlation with ADHD symptom scores, and no transfer effect of learning, as observed in the proof-of-concept study [25].

While the results of the larger study were unexpected, the smaller proof-of-concept RCT showed that fMRI-NF effects seem to be more pronounced on activation during a Stop task than on clinical and cognitive measures [20–22]. Thus, we examined the effects of active fMRI-NF versus sham fMRI-NF of rIFC on fMRI activation related to successful and failed inhibition during a tracking Stop task in boys with ADHD. We hypothesized that as in the previous study [20,21], active fMRI-NF versus sham fMRI-NF of rIFC would increase activation in the right IFC during successful stop trials and in left IFC during error monitoring. We furthermore hypothesized that these changes would be associated with improved performance in the key performance measures of inhibitory control and error monitoring, respectively, as well as with improvement in ADHD symptoms.

## 2. Methods

### (a) Trial design

In this pre-registered (ISRCTN14491589) double-blind, sham-controlled, parallel RCT, participants were block-randomized into an active or sham group with a 1 : 1 ratio and varying block sizes, stratified by medication status (non-medicated/on stable ADHD medication) and by age group (up to or over 14 years and six months). Randomization was conducted independently by the King’s Clinical Trials Unit.

Families, and researchers who were involved in data collection, were blind to the participants’ group allocation. Once a participant was allocated to a treatment arm, one researcher was unblinded to administer the treatment condition to the participant via a shielded computer terminal. This researcher, after unblinding, had no interaction with the participants/families and was strictly prohibited from sharing the information with other team members. Blinding integrity was tested by asking blinded participants, carers and researchers to guess group allocation at post-treatment and was successful for participants and their parents, but not for researchers [25]. This trial received research ethics committee approval from the UK National Health Service Health Research Authority, London Bromley Research Ethics Committee (ref. no. 17/LO/1368), was conducted in accordance with the Declaration of Helsinki 1975 and is reported in line with Consolidated Standards of Reporting Trials (CONSORT) guidelines [25].

### (b) Participants

Participants were 88 boys, aged 10–18 years, meeting the Diagnostic and Statistical Manual of Mental Disorders-5 (DSM−5 [1]) diagnostic ADHD criteria confirmed by the Kiddie Schedule for Affective Disorders and Schizophrenia interviews (KSADS; [26]), and with a *t*-score of 60 or more in the Conners 3P [27] DSM−5 inattention and/or hyperactivity-impulsivity domains. Participants were either medication-naïve or on stable ADHD medication for at least two weeks before baseline and until post-treatment assessment. Stimulant users were requested to abstain from taking medication 24 h before each of the three assessments but could remain on medication throughout the study if they wanted. Exclusion criteria were IQ less than 80 [28]; any co-occurring psychiatric disorder, except for oppositional defiant disorder and conduct disorder that is commonly co-occurring in ADHD; neurological conditions or contraindications to MRI. Only boys with ADHD were included since the selection of our NF target of rIFC was based on pediatric meta-analytic findings that included over 84% of ADHD males [7–9]. The inclusion of females in a neurotherapy study of rIFC upregulation would have been premature and not ethical given that, in the absence of fMRI meta-analyses evidence in large number of females, it is currently unknown whether girls with ADHD also have consistent rIFC underactivation. The parents/participants gave informed consent/assent. Participants received £180 to participate in the study plus travel cost reimbursement.

### (c) Procedure

**Figure 1.**
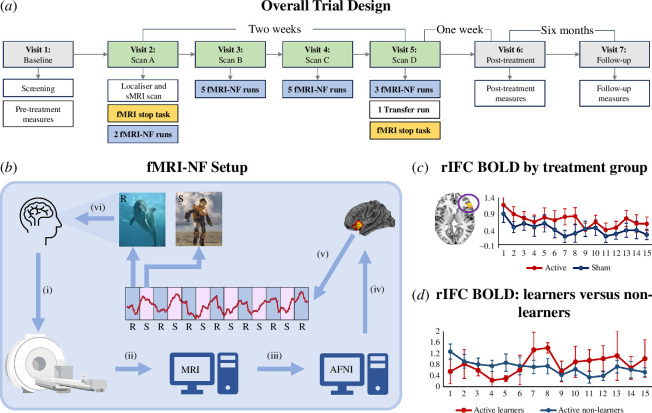
The overall trial design, fMRI-NF set-up and rIFC activation across runs. (*a*) This study was part of a larger trial assessing the efficacy of fMRI-NF for children with ADHD that consisted of seven visits including four fMRI scan sessions. The fMRI Stop task occurred during the scan visit A and D before the first fMRI-NF run and after a transfer run. (*b*) This diagram depicts the fMRI-NF architecture. Each fMRI-NF run consisted of seven rest (R) and six self-regulation (S) blocks. During the S block, (*b*(i)) the participant brain scan images were acquired in the MRI scanner, (*b*(ii)) reconstructed in the MRI station, (*b*(iii)) pre-processed using the Analysis of Functional NeuroImages (AFNI) in a remote server to compute (*b*(iv)) fMRI-NF signal that enabled (*b*(v)) control of the rocketeer that was visually fed back to the participant. (*c*) The average BOLD activation extracted from the rIFC cluster for the sham and the active groups is depicted across 15 runs. (*d*) The same signal from individuals in the active group divided into those who were judged as learners and non-learners.

Participants were invited for seven study visits, consisting of eligibility screening and baseline assessment (visit 1), fMRI-NF interventions (visits 2–5), post-treatment (visit 6), and six month follow-up assessments (visit 7) (for further details, see Lam *et al*. [25]). fMRI of the tracking Stop task was conducted at visit 2, before the first of the fMRI-NF runs and at treatment visit 5, after the 15 fMRI-NF runs (figure 1*a*).

#### (i) Functional magnetic resonance imaging neurofeedback intervention

Treatment comprised 15 active or sham fMRI-NF runs over four 1 h scan sessions. Each run consisted of seven 30 s ‘rest’ blocks and six 40 s ‘self-regulation’ blocks (i.e. 7.5 min total; figure 1*b*). During the rest blocks, the participants passively viewed a static image of a dolphin and were instructed to relax and keep still. During the self-regulation blocks of active fMRI-NF, participants were asked to move a gamified rocketeer—displayed on a screen in front of them during the scanning session—up into space by any means they could. The rocketeer’s movement was based on the activation of the opercular and triangular parts of rIFC, and participants received real-time visual feedback of their self-regulation through the rocketeer moving up or down on the screen (see a brief video showing the rocketeer game animation at [29]). After each run, participants were given a score between 1 and 10 as feedback on their upregulation performance. The participants were not given specific instructions but were told that concentrating might help self-upregulation of activation. After the last fMRI-NF run of the last session, a ‘transfer run’ was performed, which was identical to the previous runs except that no feedback was provided [25].

#### (ii) Sham condition

The sham group underwent identical procedures, except that they received sham NF during the ‘self-regulation’ blocks, i.e. the rocketeer moved according to brain activity generated by the last active participant rather than their own. The playlists of the sham NF were generated using either fully completed active NF runs or for those active participants who did not complete 15 runs (but at least eight runs), some runs were repeated to create a full playlist, randomizing their order in the playlist. A sham playlist using data from an active fMRI-NF pilot participant was created (18-year-old healthy control) in case the first study participant was randomized to the sham group (for further details see Lam *et al*. [25]).

#### (iii) Acquisition and real-time data processing for functional magnetic resonance imaging neurofeedback signal

Imaging data were acquired at the Centre for Neuroimaging Sciences, King’s College London, on a GE Discovery MR750 3T scanner (GE Medical Systems, Chicago, USA) equipped with a 12-channel head coil for signal reception. fMRI-NF scans were acquired using a T2*-weighted Echo planar imaging (EPI) sequence, sequentially from top to bottom [25]. Control of the rocketeer game was enabled by real-time transfer and analyses of fMRI data, facilitated by the Analysis of Functional NeuroImages (AFNI) software [30,31] and a custom real-time fMRI interface [32], consisting of a set of scripts installed on the MRI scanner to test connection between all computers, and to easily stop, start and monitor AFNI’s real-time fMRI software both on the scanner and on a remote Linux workstation image processing server (see the electronic supplementary material, Methods).

#### (iv) Offline processing of the functional magnetic resonance imaging neurofeedback runs

The offline processing strategy of the fMRI-NF runs is presented in the electronic supplementary material, methods and was also described fully in a previous publication [25]. The mean BOLD signal extracted from the significant cluster in the rIFC across the 15 fMRI-NF runs for the active and sham groups is presented in figure 1*c*. Among those in the active group, seven participants were categorized as ‘learners’ in an additional *post hoc* analyses, defined by a correlation of *r* > 0.15 between their rIFC brain activation and the number of runs [33], indicating progressive learning (figure 1*d*; electronic supplementary material, Results table S1).

#### (v) Functional magnetic resonance imaging neurofeedback tracking Stop task

The 6 min fMRI version of the individually adjusted visual tracking Stop task used here has been described previously [20,34–36]. Prior to scanning, each participant practised the 6 min rapid mixed trial, event-related Stop task. The task required withholding a motor response to a Go stimulus when it was followed unpredictably by a Stop signal [34–36]. The basic task was a choice reaction time task (left and right-pointing arrows: Go-signals) with a mean inter-stimulus interval of 1.8 s (156 Go trials) where subjects had to press the corresponding left or right button when viewing the left or right arrows. In 20% of the trials, pseudo-randomly interspersed, the Go-signals were followed (about 250 ms later) by arrows pointing upwards (Stop-signals), and participants had to inhibit their motor responses (in a total of 40 Stop trials). A tracking algorithm changed the time interval between Go-signal and Stop-signal onsets according to each participant’s inhibitory performance to ensure that the task was equally challenging for everyone and to provide 50% successful and 50% unsuccessful inhibition trials at every moment of the task. The task measured motor response inhibition in the 50% successful Stop trials as well as error/performance monitoring in the 50% unsuccessful Stop trials. Participants perceived direct feedback of their inhibition failure by seeing the Stop signal appear after they had made their motor response. The primary dependent behavioural variable for the motor inhibition capacity is the Stop-signal reaction time (SSRT), which was calculated by subtracting the mean delay time from the mean reaction time (MRT) to Go trials [37]. The post-error response time slowing (PERTS) was the primary dependent variable of the error monitoring process. It was an index of the awareness of one’s own inhibitory failure (i.e. an indicator of slowing down after making a mistake), which was computed by subtracting MRT to Go signals after a successful motor inhibition from MRT to Go trials after an unsuccessful motor inhibition [38]. Other dependent variables of the Go process of the task were the MRT to Go signals, intrasubject response time variability, and omission errors to Go signals.

#### (vi) Functional magnetic resonance imaging neurofeedback Stop task data acquisition

Before the first real-time fMRI-NF run and after the last fMRI-NF transfer run, functional scans for the fMRI Stop task were collected. In each of 38 non-contiguous planes parallel to the anterior-posterior commissure, 200 T2*-weighted magnetic resonance (MR) images depicting BOLD contrasts covering the whole brain were acquired with repetition time/echo time (TR/TE) = 1800/30 ms, flip angle = 75°, 64 × 64 matrix size, field of view (FOV) = 211 mm, slice thickness = 3 mm, slice gap = 0.3 mm and voxel size = 3.3 × 3.3 × 3.3 mm^3^ (voxel dimensions matching the real-time fMRI-NF runs). At the start of the first fMRI-NF session, a high-resolution T1-weighted structural sagittal ADNI GO ACC MPRAGE was also acquired with inversion time/TR/TE = 400 ms/7.312 ms/3.016 ms, flip angle = 11°, 196 slices, FOV = 270 mm, 256 × 256 matrix size and slice thickness 1.2 mm with no slice gap.

fMRI data of the participants were pre-processed using statistical parametric mapping (SPM12). The pre-processing steps included slice-time correction, realignment of EPI series to middle volume to correct head motion, co-registration with the individual’s structural T1 scan, segmentation, normalization to the Montreal Neurological Institute (MNI) EPI template and smoothing with a 6 mm Gaussian kernel.

First, we conducted data analyses at the subject-level. BOLD responses were modelled by convoluting the canonical hemodynamic response function with event onsets, while covarying for six translational and rotational motion parameters [39–41] and for volumes with frame-to-frame motion greater than 1 mm as additional spike regressors [42,43] to control for residual volume-to-volume head motion. We modelled the successful Stop trials, failed Stop trials, omission errors, and all other errors, and used the correct Go trials as an implicit baseline. A high-pass filter (128 s) was applied to reduce low-frequency noise, and a first-order autoregressive model was used to correct time series correlation. As stated in our pre-specified analysis plan [44], the two primary outcome contrasts of interest were (i) successful Stop versus Go, and (ii) failed Stop versus Go, reflecting motor response inhibition and performance/error monitoring, respectively.

Group-level analyses were conducted using the two contrasts. First, within-group brain activation associated with each contrast for the sham and the active group at pre- and post- treatment were derived using a one-sample *t*‐test at an uncorrected voxel of *p* <0.001, and family-wise error (FWE)-corrected cluster extent of *p*_FWE_ < 0.05. To investigate the effect of the fMRI-NF treatment on motor inhibition or performance monitoring, we used the flexible factorial design in SPM12, defining the main effects of group, time and the interaction of group x time as contrasts of interests, while covarying for age, medication status and total movement. The analyses were undertaken with all participants data in the first instance and repeated in sensitivity analyses excluding: (i) individuals with omissions errors of greater than 30% [45], and (ii) individuals whose total movement made them outliers (i.e. with absolute total movement value 1.5 × interquartile range beyond the first and third quartile). These analyses were conducted at the whole-brain level, with a follow-up small volume correction (SVC) applied to the left and right IFC combining opercular and triangular parts, as both regions are key areas of motor response inhibition and error monitoring [34,46–56] and were activated with fMRI-NF of rIFC in the proof-of-concept trial [20–22].

### (d) Statistical analysis

Behavioural and questionnaire data preparation and statistical analyses were conducted using IBM SPSS Software 26 (Armonk, NY). Participant characteristics pre-treatment were compared between groups (active, sham) using independent *t*‐test, chi-square test or Fisher’s exact test as appropriate. Changes in the Stop task performance measures across time between groups were analysed using a series of 2 (group) × 2 (time) repeated analyses of covariance (ANCOVAs). In these ANCOVAs, each behavioural measure (e.g. SSRT, PERTS) was entered as a dependent variable, while covarying for the participants’ age and medication status pre-treatment. The main effects of group and time, and interaction between group x time, were examined for SSRT and PERTS. The analysis was not corrected for multiple testing for the primary outcome variables, the SSRT and PERTS, but it was corrected for multiple testing for all other variables per effect (main or interaction) using the false discovery rate correction method [57]. Significant group × time interactions were explored using simple-effect analyses by extracting the mean value of the cluster activation. The exploratory simple effect analyses were conducted without multiple testing.

To investigate the relationship between brain activation and cognitive or clinical changes, correlations were conducted between the mean cluster activation values and the primary measure of cognitive function associated with the activation (e.g. activation from the contrast of failed Stop versus Go with difference in PERTS post-fMRI-NF minus pre-fMRI-NF) and change scores of ADHD-RS (i.e. post-fMRI-NF minus pre-fMRI-NF scores) in the active group. To examine the role of individual differences, these correlational analyses were also conducted separately in the sham group. In line with a previous approach, the brain-behavioural analyses were not corrected for multiple comparisons [20,21]. Finally, to examine whether performance might be associated with learning status, we compared post hoc progressive learners (*n* = 7; 15.9%) versus non-learners (*n* = 37) in their change scores using a series of independent *t*-tests.

## 3. Results

### (a) Participants

Between 2 January 2018, and 11 March 2020, 122 families completed the baseline assessment and 94 (77.0%) were randomized into active or sham groups (electronic supplementary material, S1). Six participants (6.4%) refused scanning and dropped out, leaving 44 participants per group [25]. The trial was stopped prematurely owing to the COVID-19 lockdown, reducing the sample from the target recruitment number of 100. Groups did not significantly differ at baseline (table 1), except that there were more participants with ADHD-combined presentations in the active compared to the sham group ( $\chi_{1,88}^{2}$ = 6.47; *p* = 0.011). Not all participants completed all 15 fMRI-NF runs (table 1); the number of runs completed by participants did not differ between active and sham groups (*M* = 14.2 for each group; *t*_1,86_ = 0.17; *p* = 0.87).

**Table 1.** Characteristics of the active and sham participant groups. (FSIQ, full-scale IQ; ODD, oppositional defiant disorder; CD, conduct disorder; DSM-5, Diagnostic and Statistical Manual of Mental Disorders—5th edition; SCQ, social communication questionnaire; KSADS, Kiddie Schedule for Affective Disorders and Schizophrenia; *n*, participants number; *M*, mean; s.d. = standard deviation; MPH, methylphenidate. *p*-values were uncorrected for multiple testing.)

	active	sham	statistics	
	(*n* = 44)	(*n* = 44)		
(*a*) demographics	*M* (s.d.)	*M* (s.d.)	*t*_86_	*p*-values
age, in months	13.1 (7.30)	13.3 (8.14)	0.46	0.65
education,in years	8.05 (1.93)	8.30 (2.32)	0.55	0.58
FSIQ	102.4 (13.6)	104.8 (12.4)	0.88	0.38
(*b*) dimensional trait measures	*M* (s.d.)	*M* (s.d.)	*t*_86_	*p*-values
ADHD-RS				
total score	37.3 (9.5)	37.8 (9.3)	0.25	0.8
inattention	19.8 (4.4)	21.3 (4.0)	1.76	0.08
hperactivity/impulsivity	17.5 (6.0)	16.5 (6.8)	−0.78	0.44
Conners−3P				
DSM−5 Inattention	80.0 (8.3)	81.1 (7.7)	0.67	0.51
DSM−5 hyperactivity/ impulsivity	84.3 (10.1)	81.4 (13.7)	−1.11	0.27
DSM−5 ODD	72.5 (14.3)	69.3 (15.1)	−1	0.32
DSM−5 CD	59.6 (14.1)	57.7 (16.1)	−0.58	0.57
ADHD Index	14.5 (4.36)	13.5 (4.2)	−1.05	0.3
SCQ	7.50 (4.63)	6.98 (5.93)	0.46	0.65
(*c*) KSADS diagnostic measures	*n* (%)	*n* (%)	*χ* ^2^ ^a^/FET^b^ *p*	
ADHD research diagnosis				
combined presentation	36 (81.8)	25 (56.8)	0.01^a^	
inattentive presentation	8 (18.2)	19 (43.2)		
ODD	21 (47.7)	18 (40.9)	0.52^a^	
CD	1 (2.3)	0	>0.99^b^	
alcohol use	1 (2.3)	0	>0.99^b^	
drug use	0	0	--	
(*d*) medication	*n* (%)	*n* (%)	FET^b^ p	
medication status				
naïve	11 (25.0)	14 (31.8)	0.88^b^	
currently medicated—off	15 (34.1)	15 (34.1)		
currently medicated—on	14 (31.8)	12 (27.3)		
not currently medicated	4 (9.1)	3 (6.8)		
current medication type				
no medication	11 (25.0)	14 (31.8)	0.62^b^	
methylphenidate	24 (54.5)	26 (59.1)		
(Lis)dexamfetamine	6 (13.6)	3 (6.8)		
atomoxetine	2 (4.5)	1 (2.3)		
MPH and guanfacine	1 (2.3)	0		
(*e*) fMRI-NF	*M* (s.d.)	*M* (s.d.)	*t*_86_	*p*-values
completed number of runs	14.2 (1.2)	14.2 (1.3)	0.17	0.87

^a^ *p*-values for *X* ^2^ statistics.

^b^ *p*-values for Fisher’s exact test (FET).

### (b) Group differences in treatment-related changes in functional magnetic resonance imaging Stop-task performance

The probability of inhibition was approximately 50% for both the active (*M*_PRE_ ± s.d. = 50.6 ± 3.46; *M*_POST_ ± s.d. = 51.7 ± 8.57) and the sham group (*M*_PRE_ ± s.d. = 51.3 ± 6.15; *M*_POST_ ± s.d. = 53.0 ± 6.72), indicating that the tracking mechanism of the fMRI stop task was operational. Probability of inhibition did not differ between groups (*F*_1,86_ = 0.26, *p* = 0.61).

**Figure 2.**
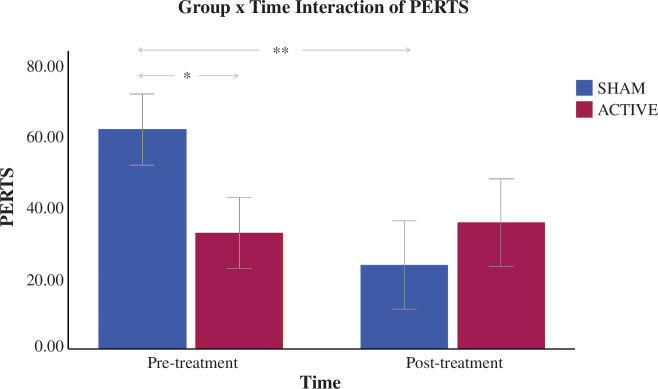
Group × time Interaction of post error response time slowing (PERTS). Simple-effect analyses showed a significantly higher PERTS in the sham versus active group at pre-treatment (*p* = 0.043) and a decrease of PERTS in the sham group from pre- to post-treatment (*p* = 0.007). Error bars indicate the standard error of the mean. Significant thresholds: *p < 0.05, **p < 0.01.

Repeated 2 × 2 ANOVA analyses revealed a significant effect of time (*F*_1,86_ = 4.47, *p* = 0.038) with a significant group x time interaction (*F*_1,86_ = 4.37, *p* = 0.040) for PERTS (table 2). Simple-effect analyses showed significantly higher PERTS in the sham relative to the active group at pre-treatment (*p* = 0.043) but not at post-treatment (*p* = 0.50), and a significant pre- to post-treatment reduction of PERTS in the sham (*p* = 0.007) but not the active group (*p* = 0.84) (figure 2).

**Table 2.** Stop task performance by group pre- and post-treatment. (ANCOVA, analysis of covariance; PRE/POST, pre-/post-treatment; *M*, mean, s.d., standard deviation; SSRT, stop-signal reaction time; PERTS, post-error response time slowing; MRT, mean reaction time; RTV, response time variability.)

	active		sham		repeated 2 × 2 ANCOVA					
	PRE	POST	PRE	POST	group		time		group x time	
	*M* (s.d.)	*M* (s.d.)	*M* (s.d.)	*M* (s.d.)	*F*_1,86_	*p*	*F*_1,86_	p	*F*_1,86_	*p*
SSRT	179.6 (124.8)	150.1 (163.3)	173.8 (144.4)	105.9 (144.6)	0.57	0.45	0.15	0.7	1.74	0.19
PERTS	32.6 (64.3)	34.7 (92.1)	61.6 (73.5)	24.3 (68.2)	0.48	0.49	4.47	0.038	4.37	0.04
MRT to Go	637.6 (126.4)	677.8 (135.3)	655.3 (108.4)	657.9 (118.0)	<0.001	0.98	0.37	0.55	3.17	0.079
intrasubject RTV	178.2 (44.0)	189.8 (53.6)	181.8 (40.8)	180.3 (36.9)	0.18	0.67	2.42	0.12	1.4	0.24
omission errors	6.2 (7.80)	5.91 (6.85)	7.91 (11.2)	7.23 (9.13)	3.25	0.34	0.016	0.9	0.044	0.84

### (c) Within-group contrasts of successful Stop versus Go and failed Stop versus Go

**Figure 3.**
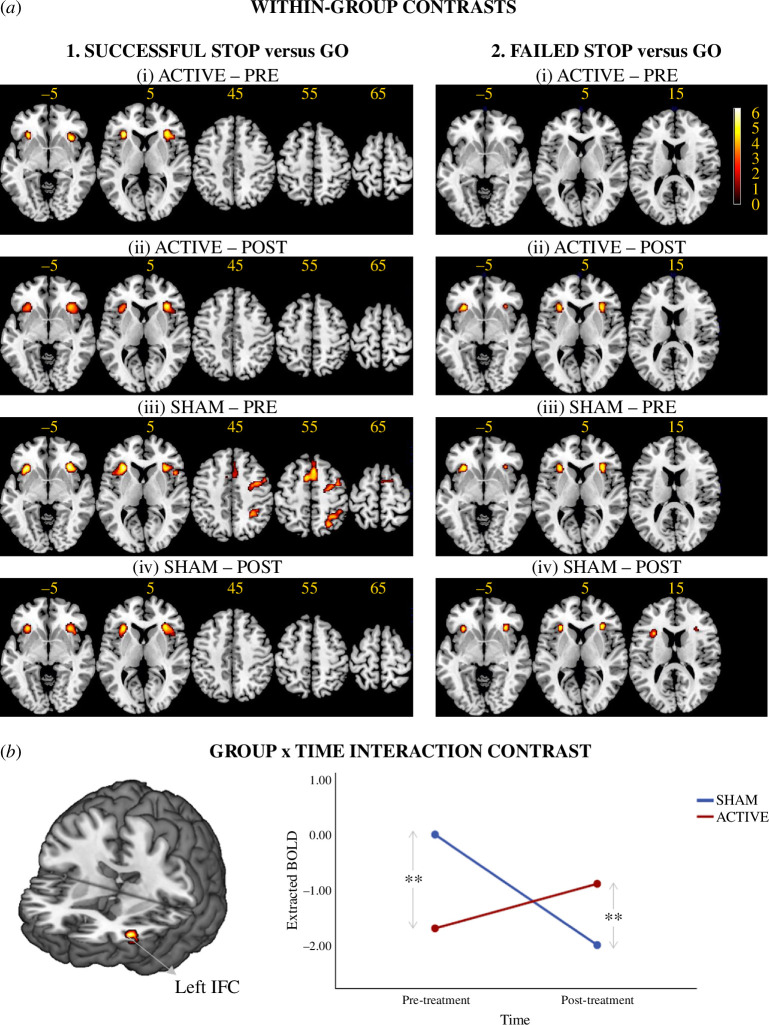
Within-group and group x time interaction brain activation clusters. (*a*) Within-group activation clusters, uncorrected at peak *p* < 0.001 and family-wise corrected cluster level *p*_FWE_ < 0.05, associated with (i) successful Stop versus Go trials, and (ii) failed Stop versus Go trials in the active and sham group at pre- and post-treatment scans. The Go trials were modelled as an implicit baseline. (*b*) Group x time interaction revealed significant clusters at the left IFC after small volume correction at the region of left IFC combined opercular and triangular parts at uncorrected peak *p* < 0.001.

The contrast successful Stop versus Go (with Go as implicit baseline) evoked significant activation clusters in the active group in bilateral anterior insula (AI)/orbital IFC during pre-treatment, extending to bilateral ventrolateral prefrontal cortex (vlPFC) during post-treatment. The contrast evoked activation in the sham group during pre-treatment in bilateral AI/orbital IFC/vlPFC extending to right IFC triangular/opercular parts/precentral/middle frontal cortices, as well as right inferior/superior parietal lobe, and supplementary motor cortex extending to right superior frontal cortex; and during post-treatment in bilateral AI/orbital IFC/vlPFC only (figure 3*a-*1).

The contrast failed Stop versus Go (with Go as implicit baseline) evoked no activation clusters in the active group pre-treatment but evoked activation post-treatment in bilateral AI/orbital IFC. The same contrast evoked clusters of activation in bilateral AI/orbital IFC in the sham group during both the post- and pre-treatment scans (figure 3*a*(ii)).

### (d) Group differences in treatment-related changes of functional magnetic resonance imaging activation

Total movement did not differ significantly between groups (*F*_1,86_ = 0.16, *p* = 0.90), nor did it change significantly across time points (*F*_1,86_ = 2.50, *p* = 0.12). No interaction between group x time was evident (*F*_1,86_ = 0.35, *p* = 0.56) at the whole-brain level. Total movement was included as a covariate in the model to remove residual within-group motions.

The flexible factorial model, covarying for total movement, age and medication status revealed no brain activation clusters reflecting significant effects of group, time or interaction of group x time for the contrast successful Stop versus Go neither in the whole-brain analysis nor in the SVC analysis in left or right IFC region only. The same model applied to the contrast failed Stop versus Go equally showed no activation clusters in the whole-brain or the SVC analysis of rIFC. However, the SVC applied on the left IFC revealed a significant cluster associated with a group x time interaction (*p* = 0.034, *F* = 20.1, MNI peak coordinates (*x* = −40, *y* = 44, *z* = 6), cluster size (*k*_E_) = 47 voxels; figure 3*b*), which remained significant after removing individuals who had more than 30% omission errors to Go trials (*p* = 0.029, *F* = 20.4, (−40, 44, 6), *k*_E_ = 53 voxels) and after additionally removing individuals who were outliers in terms of excessive movement (*p* = 0.048, *F* = 21.3, (−40, 44, 6), *k*_E_ = 35 voxels). Simple effect analyses of extracted data from the cluster showed significant differences between the active and sham at pre- (*p* = 0.002) and post-treatment (*p* = 0.009) and a significantly reduced activation in the cluster for the sham group (*p* < 0.001), and a weak, although not significant, increase of activation in the active group (*p* = 0.076). *Post hoc* exploration at a more lenient threshold for peak *p* < 0.01 (uncorrected) and FWE-corrected cluster-extent *p*_FWE_ < 0.05, showed extension of the left IFC cluster frontally to left dorso-, rostro and orbito-frontal PFC (electronic supplementary material, Results S2).

### (e) Brain-behavioural relations

Correlations were not significant between the cluster of brain activation associated with the significant group x time interaction in the left IFC and the change scores of PERTS (*r*_p_ = 0.17, *p* = 0.28) and ADHD-RS total (*r*_p_ = −0.13; *p* = 0.42), inattentive (*r*_p_ = −0.19, *p* = 0.22) and hyperactive scores (*r*_p_ = −0.051, *p* = 0.75) in the active group, nor with change scores of PERTS (*r*_p_ = −0.008, *p* = 0.96) and ADHD-RS total (*r*_p_ = 0.018; *p* = 0.91), inattentive (*r*_p_ = 0.11, *p* = 0.48) and hyperactive scores (*r*_p_ = 0.070, *p* = 0.66) in the sham group.

### (f) Behavioural change comparisons between learners and non-learners

Although mean values of PERTS and ADHD-RS total and subdomain change scores are in the favourable direction for the active learner group, no differences were found between learners and non-learners in these variables (0.31<*p*s <0.98; electronic supplementary material, Results table S2).

## 4. Discussion

In this study, we tested the effects of 15 runs of active fMRI-NF versus sham fMRI-NF of rIFC on the fMRI activation and performance related to successful and failed inhibition during a tracking Stop task in 88 children and adolescents with ADHD. We found no significant effects of active fMRI-NF versus sham fMRI-NF of rIFC on inhibitory task performance nor on rIFC activation during successful inhibition. We found, however, a significant group x time interaction effect both for the post-error reaction time slowing effect and on left IFC activation during error monitoring in the small volume correction analyses focused on inferior prefrontal regions.

The finding of no treatment-related effect of fMRI-NF of rIFC on the activation of rIFC during the successful Stop condition was unexpected, given that our proof-of-concept study in ADHD adolescents has shown significant upregulation in rIFC associated with successful Stop after 11 runs of fMRI-NF of rIFC when contrasted with active fMRI-NF of the PHG [20]. Differences in trial design could account for the differences in findings. For instance, here we compared 15 runs of 7.5 min of fMRI-NF of rIFC instead of 11 runs of 8.5 min in the previous study, but it is unlikely that the number or timing of the runs, which was not sufficiently different between two studies, accounts for the finding differences.

One key difference between this study and the previous one, is the control condition. Here, the control condition was sham fMRI-NF of the rIFC, while in the previous study it was active fMRI-NF in the left PHG, which is a posterior and left-hemispheric region. The contrast of fMRI-NF of rIFC with fMRI-NF of a different brain region—as opposed to sham fMRI-NF of the same region—may have enhanced the differential NF learning effects in both groups. We observed in the previous trial that while the rIFC-fMRI-NF group showed progressively increased activation in rIFC after eight runs, activation in rIFC was progressively reduced in the PHG-fMRI-NF group after eight runs, and vice versa (i.e. PHG increased in the PHG-fMRI-NF group and decreased in the IFC-fMRI-NF group). In the study, however, we observed overall increased activation in rIFC in the active fMRI-NF group relative to the sham fMRI-NF group, while the sham group also activated rIFC, albeit in a more ventral location [25]. Furthermore, while there was overall increased activation in the active fMRI-NF versus sham fMRI-NF group in rIFC when combined across all runs (as well as in other frontal, temporal and occipital regions), there was no progressively increased activation across runs in rIFC, indicating no progressive learning effect [25].

It has been shown that the choice of contrasting conditions is critical for the outcome of fMRI-NF [58]. Sham fMRI-NF has been shown to elicit activation in cognitive control regions [59], presumably because participants concentrate on the stimulus and try to upregulate activation, including the rIFC, a key cognitive control region, even if they are not successful because they receive incorrect feedback. It is, therefore, possible that our sham control condition, given that it also elicited activation in the ventral rIFC, a crucial activation cluster for motor response inhibition, cancelled out potential effects of rIFC fMRI-NF training on rIFC activation. Regions not involved in self-control and feedback monitoring may hence be a better choice of control conditions than sham fMRI-NF [17,58,60].

Other explanations include the difference in participants’ age and medication status. In this study, participants were younger, between 10 and 18 years old, with an average age of 13 years compared to the average age of 14 years in the previous trial, which was in adolescents between 12 and 18 years [20–22]. Evidence suggests that fMRI-NF learning may be less efficient in younger participants and in those with more severe ADHD symptoms, which are characteristics of lower age [61]. Furthermore, most participants (i.e. 65%) were taking stimulant medication. This could have masked potential NF-related rIFC upregulation effects, given that stimulants have shown to consistently increase activation in rIFC both after acute and chronic dosages [8,9,13]. However, the proportion of stimulant-treated adolescents with ADHD in the previous trial was even higher (77%) and yet we still observed increased rIFC activation during successful inhibition in the Stop task [20]. Alternatively, fMRI-NF may interact with medication, which could explain why findings were positive in the previous trial where a higher proportion of patients were medicated [20]. The negative findings of no improvement in successful Stop-task activation parallel the negative findings of no rIFC fMRI-NF effects on clinical or cognitive performance [25].

Importantly, in an additional post hoc examination, we found a lower number of rIFC fMRI-NF progressive learners relative to those in the previous trial (15.9 versus 44.4% [33]). The number or learners of fMRI-NF varies across studies (25–75%, e.g. [62–67]), which might be attributed to factors such as participant characteristics, target regions, or the definition of learners (i.e. based on the slope of learning curves or last versus first run [17]). In EEG-NF, an estimated 15–30% individuals cannot operate NF accurately [68]. For individuals with ADHD, successful learning of EEG-NF across definitions have been reported in up to 50% of participants [69–71], with a study not identifying learning at a group level [72]. Across these fMRI-NF and EEG-NF studies, therefore, it is common to find a high proportion of non-learners that seems to vary substantially.

Despite the relatively low number of learners, we found a significant group-by-time interaction effect of active fMRI-NF versus sham fMRI-NF of rIFC on the activation in left IFC during error monitoring, in the failed Stop trials, as well as on the associated performance measure of post-error slowing. This replicates the findings of our previous trial where we also observed increased activation in the rIFC fMRI-NF group compared to the active PHG fMRI-NF control group after compared to before treatment in a similar-sized cluster in left IFC, which reached ventrally to the insula, premotor cortex and putamen during failed Stop trials [21]. Simple-effect analyses showed that the decrease in the sham group from pre- to post-treatment was larger and significant compared to the more marginal increase in the active group (figure 3), which appears to have driven the group by time interaction effects. This activation was significantly greater in left IFC in the sham group relative to the active group before treatment and reversed to become significantly lower in the active group relative to the sham group after treatment. The significant group-by-time effect for post-error slowing replicated the findings of our previous trial [21]. In the context of this study, it was entirely owing to the sham group decreasing in post-error slowing after relative to before treatment, while the active group appeared to show no significant differences of post-error slowing from pre- to post-treatment, although its mean value was in the expected direction.

In typically developing individuals, there is usually a performance adjustment after mistakes, reflected by slowing down in the following trials. This is thought to be a combination of self-monitoring (error detection/awareness) and adaptive control (behaviour adjustment) [51,73–77]. Children and adults with ADHD usually have difficulties in error detection/performance monitoring and do not slow down after mistakes, which has been observed in many cognitive tasks [49,75,77,78], including in motor inhibition in the Go/No-go [74] and Stop tasks [73]. ADHD has thus been associated with difficulties in error awareness [79] and it has even been hypothesized that the self-regulation difficulties in ADHD could be owing to impaired error awareness [77].

While successful inhibition has been associated more prominently with rIFC [34,46–48], left IFC has more commonly been linked to failed inhibition and error monitoring, together with other error-monitoring areas such as the anterior cingulate cortex (ACC)/mesial frontal cortex/supplementary motor area (SMA), anterior insula, and basal ganglia [34,47,49–56]. During error monitoring, individuals with ADHD have shown underactivation of left IFC as well as other regions such as rIFC, left dorsolateral prefrontal cortex (which was also found in the *post hoc* exploration in this study), pre-SMA, posterior parietal and thalamic areas [24,35,40,43]. While the medial prefrontal cortex/ACC seems to be responsible for action monitoring and serves as an alarm after making mistakes, the left IFC seems to be in charge of the cognitive control aspect by reallocating attentional resources and increasing the motor threshold [80–82], and hence appears to implement the behaviour adjustment following an error [83]. While both components of error monitoring have been found to be lower in ADHD children [24,35,77], our findings suggest that fMRI-NF of rIFC improves the mechanism underlying the *control* rather than the *action monitoring* aspects of error monitoring.

The small increase in activation in the left IFC with fMRI-NF of the rIFC, compared to sham NF, is particularly noteworthy because stimulant medication has been demonstrated to increase, and even normalize, activation in both the left and right IFC, as well as in other error-monitoring regions, such as the insula, basal ganglia and parietal regions [24]. It thus appears that fMRI-NF of rIFC has a similar effect as stimulants in increasing activation in the left IFC error monitoring region [25]. The increase of left IFC activation during error monitoring with fMRI-NF of rIFC could suggest self-upregulation of an isolated prefrontal region that has a more widespread effect on other frontal systems in ADHD, in this case of a left homologue region mediating associated self-control and self-monitoring functions, which replicates our previous finding of left IFC upregulation during error monitoring in the same task during fMRI-NF of rIFC [22].

Unlike in our proof-of-concept study, we found no significant correlation between fMRI-NF induced clinical changes and changes in left IFC in the overall active group. This could also be related to the low number of learners in this study. Note that ADHD-RS and PERTS change scores between learners and non-learners did not differ, although they were in the expected direction. However, there were too few learners for a statistically powered comparison. Altogether, considering that we found no effects of active vs sham fMRI-NF on clinical or cognitive measures, nor on brain activation during successful stop trials, the findings suggest that the current fMRI-NF of rIFC set-up has a very limited effect on a very specific brain area that mediates the cognitive control aspects of performance monitoring.

Our rigorous double-blind sham-control RCT remains, to our knowledge, the largest investigation on the effects of fMRI-NF on symptoms and brain activation for children with ADHD to date. Limitations of the study include the involvement exclusively of boys with ADHD which constrains the generalizability of findings to females with ADHD. Future neuroimaging studies should more thoroughly investigate the underlying neurofunctional differences in females with ADHD and use these as targets to develop neurotherapeutics in the female population. The sham group, relative to the active group, showed increased left IFC activation and higher PERT at baseline, which could have influenced the findings. The inclusion of mostly medicated participants could have confounded or masked fMRI-NF effects, and, despite the sample size, the study might still be underpowered for detecting smaller effect sizes. The surprisingly low proportion of progressive learners in the current study raises the need to develop fMRI-NF intervention designs that can shape the learning trajectory more optimally, which should be addressed in future fMRI-NF intervention for ADHD.

## 5. Conclusions

Our results show that active fMRI-NF relative to sham fMRI-NF of rIFC had no effect on rIFC activation during successful motor inhibition. However, it was associated with a significant group by time interaction effect on left IFC activation, owing to a small increase in the active and a significant decrease in the sham group after compared to before the treatment. The upregulation of the left IFC activation after active fMRI-NF versus sham fMRI-NF of rIFC is in line with our previous findings after fMRI-NF of rIFC relative to active fMRI-NF of PHG region [21]. The upregulation effect on left IFC is also similar to the effects of the gold-standard, stimulant medication treatment on the same region [24], but with the advantage that fMRI-NF of rIFC has no known side effects [25]. The findings show that fMRI-NF of a particular self-control target region has wider regional effects extending to another self-regulatory region, such as, in this case, the typically functionally impaired left IFC during error monitoring in ADHD. This was found despite the low number of progressive fMRI-NF learners in the current study.

## Data Availability

The data used in the current study is accessible from [84]. A brief version of the video of the rocketeer game is accessible from [85]. Supplementary material is available online [86].
